# Agent-based modeling of autophagy reveals emergent regulatory behavior of spatio-temporal autophagy dynamics

**DOI:** 10.1186/s12964-014-0056-8

**Published:** 2014-09-10

**Authors:** Christoph S Börlin, Verena Lang, Anne Hamacher-Brady, Nathan R Brady

**Affiliations:** Systems Biology of Cell Death Mechanisms, German Cancer Research Center (DKFZ), Heidelberg, Germany; Department of Surgery, Heidelberg University Hospital, Heidelberg, Germany; Bioquant, Heidelberg University, INF 267, 69120 Heidelberg, Germany; Lysosomal Systems Biology, German Cancer Research Center (DKFZ), Heidelberg, Germany

**Keywords:** Agent-based modeling, Autophagy, Autophagic flux, Cell-to-cell variability, Systems biology, Computational biology, mTOR, Lysosomes

## Abstract

**Background:**

Autophagy is a vesicle-mediated pathway for lysosomal degradation, essential under basal and stressed conditions. Various cellular components, including specific proteins, protein aggregates, organelles and intracellular pathogens, are targets for autophagic degradation. Thereby, autophagy controls numerous vital physiological and pathophysiological functions, including cell signaling, differentiation, turnover of cellular components and pathogen defense. Moreover, autophagy enables the cell to recycle cellular components to metabolic substrates, thereby permitting prolonged survival under low nutrient conditions. Due to the multi-faceted roles for autophagy in maintaining cellular and organismal homeostasis and responding to diverse stresses, malfunction of autophagy contributes to both chronic and acute pathologies.

**Results:**

We applied a systems biology approach to improve the understanding of this complex cellular process of autophagy. All autophagy pathway vesicle activities, i.e. creation, movement, fusion and degradation, are highly dynamic, temporally and spatially, and under various forms of regulation. We therefore developed an agent-based model (ABM) to represent individual components of the autophagy pathway, subcellular vesicle dynamics and metabolic feedback with the cellular environment, thereby providing a framework to investigate spatio-temporal aspects of autophagy regulation and dynamic behavior. The rules defining our ABM were derived from literature and from high-resolution images of autophagy markers under basal and activated conditions. Key model parameters were fit with an iterative method using a genetic algorithm and a predefined fitness function. From this approach, we found that accurate prediction of spatio-temporal behavior required increasing model complexity by implementing functional integration of autophagy with the cellular nutrient state. The resulting model is able to reproduce short-term autophagic flux measurements (up to 3 hours) under basal and activated autophagy conditions, and to measure the degree of cell-to-cell variability. Moreover, we experimentally confirmed two model predictions, namely (i) peri-nuclear concentration of autophagosomes and (ii) inhibitory lysosomal feedback on mTOR signaling.

**Conclusion:**

Agent-based modeling represents a novel approach to investigate autophagy dynamics, function and dysfunction with high biological realism. Our model accurately recapitulates short-term behavior and cell-to-cell variability under basal and activated conditions of autophagy. Further, this approach also allows investigation of long-term behaviors emerging from biologically-relevant alterations to vesicle trafficking and metabolic state.

**Electronic supplementary material:**

The online version of this article (doi:10.1186/s12964-014-0056-8) contains supplementary material, which is available to authorized users.

## Background

### The autophagy pathway

Macroautophagy (hereafter referred to as autophagy) is a catabolic process by which intracellular components such as proteins and organelles are delivered to lysosomal degradation, which permits the cell the ability to maintain energetic homeostasis during nutrient deprivation (ND) [1]. The autophagy pathway can be divided in four distinct steps. (1) A double-membrane structure, the phagophore nucleates in the cytosol. (2) Thereafter, the phagophore expands and encloses cellular proteins and organelles within a double-membrane organelle, the autophagosome. (3) The autophagosome then fuses with a lysosome to form an autolysosome. (4) Here, the autophagosome and its sequestered contents are degraded by lysosomal hydrolases (for a review see [2]).

Autophagy is negatively regulated by the anabolic PI3K/Akt/mTOR signaling pathway. Growth factors and amino acid abundance promote mTOR activity, which suppresses autophagy to a basal level [3]. Cellular stresses, including hypoxia [4] and low levels of energy/amino acids [5], result in mTOR inactivation, and the resulting activation of autophagy through Beclin-1 activation of the Class III PI3K, Vps34, coordinating the nucleation and formation of autophagosomes within the cytosol [2]. Autophagosomes target cytosolic components through ordered bulk degradation [6], or selective targeting by autophagy receptors [7-9].

### Spatial regulation of autophagy

An emerging body of evidence demonstrates that the regulation of individual steps within the autophagy pathway occurs locally, at subcellular compartments. Amino acids signal mTOR activation from within the lysosomal lumen [10], but also through cytosolic amino acid sensing [11,12]. In addition, components of the autophagy process are distributed in a heterogeneous and dynamic manner. While autophagosome formation occurs throughout the cell, matured autophagosomes are transported on microtubules in a dynein-dependent manner towards the nucleus [13]. Another important spatial aspect of autophagy concerns lysosomal positioning within the cell, which is affected by nutrient availability. Starvation conditions promote lysosomal clustering at the nucleus, whereas restoration of nutrient levels leads to reordering of the lysosomes towards the plasma membrane [14,15]. ND therefore influences autophagy at the levels of pathway regulation and vesicle positioning. On one hand, ND increases both phagophore and lysosome formation via the inhibition of mTOR [16]. On the other hand, ND impacts the amount of fusion events occurring between lysosomes and autophagosomes by reordering lysosomes towards the cell center, and therefore bringing autophagosomes and lysosomes into close proximity.

### Computational modeling of cellular processes

Systems biology models of a cellular process allow for dynamic exploration of biological findings and the identification of non-intuitive emergent system behavior [17]. The most common systems biology approach to pathway modeling uses ordinary differential equations (ODE) to explicitly describe component interactions and cellular processes. ODEs are solved to predict model component behaviors, in terms of concentration changes over time. An alternative approach is agent based modeling (ABM), which relies on a predefined logical programming language to implement source code representing cellular processes within a software framework. With an ABM approach one can make use of the advantages of a programming language, such as loops and other control structures, self-defined functions and hierarchical ordering of procedures, in order to create an implicit and robust description of cellular processes. Based on protein and second messenger interactions, ODE modeling has been used to predict dynamics of autophagosome behavior under varied autophagy activity states [18] and in response to apoptotic stimuli [19], with model predictions in accordance with experimental measurements. However, ODE modeling assumes a ‘mixed-bag’ environment, and cannot account for non-homogenous distributions of model components. ABMs can simulate temporal and spatial evolution of a system, where each participant in the model is represented as an individual agent following its own rule set, which encodes characteristics that determine behavior and interaction with other agents. Emergent behavior results from the individual behavior of each agent, and spatio-temporally determined interactions among agents [20]. While ABMs are commonly applied in non-biological modeling [20], recently studies using ABMs have captured population-level emergent behavior, including mitochondrial fusion and fission events [21] and apoptotic death receptor dynamics [22], thereby demonstrating the benefit of including spatial information.

### Study rationale

Autophagy is required for maintaining cellular homeostasis, and dysfunction at different steps of autophagy is causative in both chronic diseases, including cancers and different neurodegenerative disorders [23], and acute diseases, including cardiac and neuronal ischemic injuries [24]. As such, strategies to experimentally target different autophagy steps is subject of intense study [25-28].

Here we first established an ABM of the autophagy pathway based on prior knowledge and the incorporation of high-resolution fluorescence microscopy data. Through an iterative process of model improvement, via optimized fitting of data from quantified, single-cell images of autophagy, we investigated the relationship between spatio-temporal events and autophagic flux imbalances. Through simulations and experimental investigations, this approach revealed that applying a lysosomal inhibitor used to interfere with autophagic flux, rapidly (minutes to hours) resulted in mTOR inhibition. Furthermore, we demonstrate that accurate spatio-temporal modeling of autophagy required increasing model complexity, by integrating functional autophagy with the cellular nutrient state. The resulting model recapitulates with high accuracy the observed short-term behavior of autophagic flux under different conditions, including the cell-to-cell variability, and is capable of addressing long-term behaviors corresponding to biologically-relevant, minor alterations to vesicle transport and metabolic state.

## Results

### Agent-based model of autophagosome formation and degradation by lysosomes

**Figure 1:**
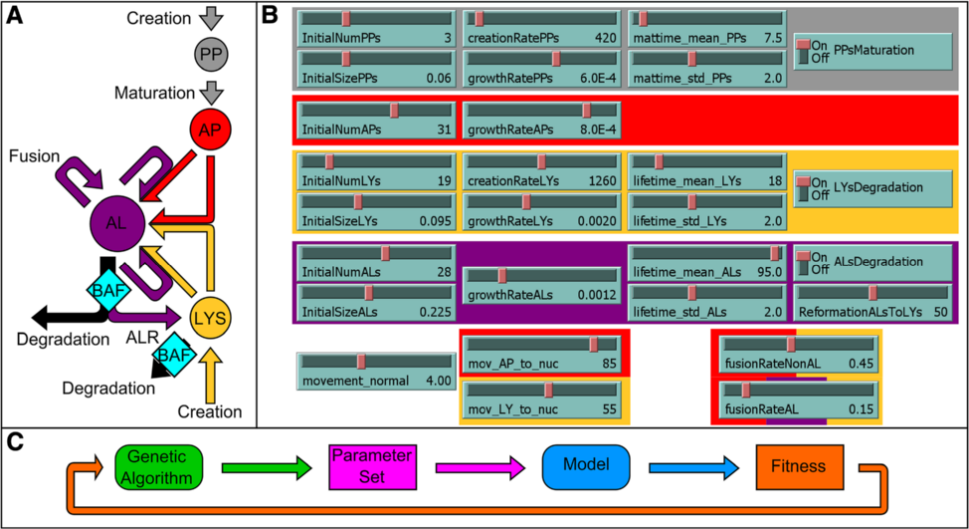
**Overview of the core model of autophagy. A** Schematic describing interaction of the four agent types. Each agent is color-coded; phagophores (PP) in grey, autophagosomes (AP) in red, lysosomes (LYS) in yellow and autolysosomes (AL) in purple. Merging arrows represent fusion events between the two agents to form or expand an autolysosome. Arrows marked with BAF indicate inhibition by Bafilomycin A1. Black arrows represent degradation events. **B** Overview of the ABM parameter set. Agent parameter colors correspond to the color code used in **A**. **C** Schematic of the implemented fitting process to find the best parameter set, performed using a genetic algorithm in combination with a predefined fitness function.

**Figure 2:**
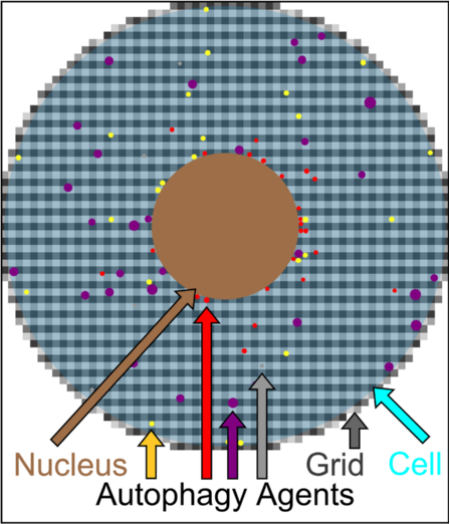
**Implementation of the core model of autophagy.** Schematic overview of the modeled cell as a cyan circle with a 30 μm diameter, including the nucleus with a 10 μm diameter, shown in brown. The implemented 0.5 μm × 0.5 μm grid is represented by the grey squares in the background. The four autophagic agent types are color-coded as follows; phagophores in grey, autophagosomes in red, lysosomes in yellow and autolysosomes in purple.

Using the NetLogo ABM platform [29], we first constructed a core model of autophagy, conceptualized as procedures describing 4 agents. The process starts with the formation of a phagophore (PP) which then grows and matures into an autophagosome (AP). This autophagosome then fuses with a lysosome (LY) to generate an autolysosome (AL). The newly formed autolysosomes can then either fuse with lysosomes, autophagosomes or other autolysosomes to grow. During these processes the autolysosome reaches its maximum lifetime, and then is degraded and removed from the system. To simulate organelle movements we assumed random motion for phagophores and autolysosomes, while autophagosomes and lysosomes move directly towards or directly away from the nucleus to mimic their active transport along the cytoskeleton, with a speed that is independent of its size [30]. The resulting model schematic, describing the 4 different autophagic agents and their possible actions, is shown in Figure 1A. The corresponding model parameters are shown in Figure 1B and Figure 1C displays the fitting process implemented. The core autophagy NetLogo Model is available in the Supplementary Information, Additional file 1. The cell was modeled as a circle with a 30 μm diameter consisting of a grid of 0.5*0.5 μm cytosolic areas. For spatial realism, the nucleus was included as a circle with a 10 μm diameter, as shown in Figure 2. Time steps (dt) are modeled in 1 minute increments.

### Single-cell quantification of autophagy under growth and nutrient deprivation conditions

**Figure 3:**
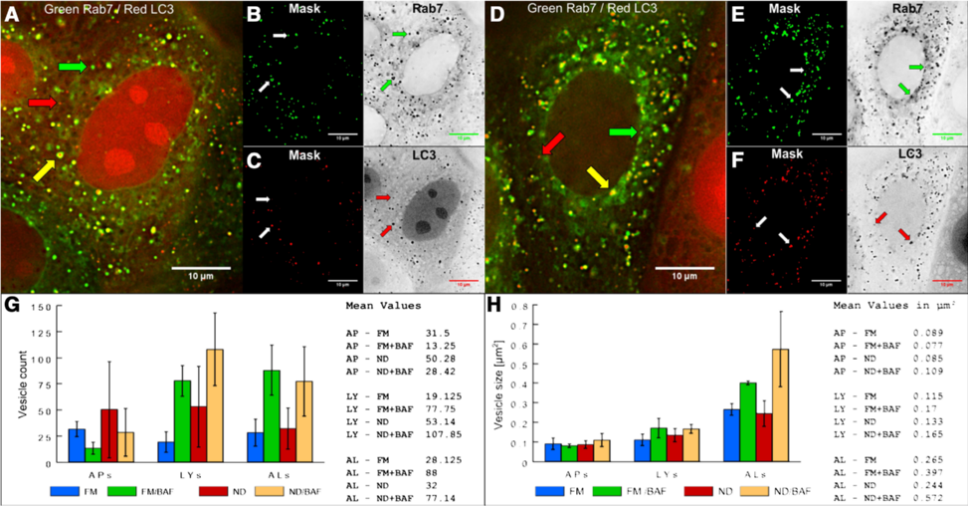
**Single cell analysis of autophagic flux in MCF7 cells.** MCF7 cells stably co-expressing GFP-Rab7 (green) and mCherry-LC3 (red) were submitted to FM and ND conditions for 3 hours, in the absence and presence of BAF (100 nM). **A** Merged image of a typical cell under FM conditions. **B**-**C** Individual green and red channels of **(A)** are shown as inverted black-white images and corresponding segmented masks. **D** Merged image of a typical cell under FM conditions with BAF inhibition of lysosomes. **E**-**F** Individual green and red channels of **(D)** are shown as inverted black-white images and corresponding segmented masks. **A**-**F** Green arrows indicate Rab7(+)/LC3B(-) endo-lysosomes, red arrows indicate Rab7(-)/LC3B(+) autophagosomes, and yellow arrows indicate Rab7(+)/LC3B(+) autolysosomes. **G** Histogram displaying APs, LYs, ALs vesicle numbers for the different conditions (left), and list of the corresponding mean values (right). **H** Histogram displaying APs, LYs, ALs vesicle sizes for the different conditions (left), and list of the corresponding mean values (right). Number of analyzed cells: FM 8, FM/BAF 4, ND 14, ND/BAF 7.

In order to parameterize our model, we measured autophagy activity steps in MCF7 breast cancer cells, at 3 hours under full medium (FM) conditions (basal autophagy), and at 3 hours of ND conditions (activated autophagy). Bafilomycin A1 (BAF, 100 nM), an inhibitor of the lysosomal v-ATPase [31] was applied in order to reveal autophagic flux [32]. We detected endo-lysosomal signaling using GFP-Rab7 [33], which participates in the fusion between lysosomes, autophagosomes and autolysosomes and is therefore located in lysosomal and autolysosomal membranes [2]. We detected autophagosomes using mCherry-LC3B, a main component of autophagosomal and autolysosomal membranes [34]. A representative image of cells under FM conditions is shown in Figure 3A-C, and a representative image under FM conditions with the addition of BAF is shown in Figure 3D-F. From segmented single-cell image masks, we identified Rab7(+)/LC3B(-) vesicles as endo-lysosomes (green arrows), Rab7(-)/LC3B(+) vesicles as autophagosomes (red arrows), and Rab7(+)/LC3B(+) vesicle as autolysosomes (yellow arrows). For all conditions and vesicles types, we calculated vesicle count (Figure 3G) and vesicle size (Figure 3H).

Our results demonstrate that autophagy responses varied from cell-to-cell, most pronounced under ND conditions. Notably, ND increased autophagosomal count approximately 1.5 fold, increased lysosomal count approximately 2 fold, and slightly increased autolysosomal count, but did not increase vesicle sizes. This increase in vesicle counts under ND conditions, i.e. under activated conditions of autophagy, showed the acceleration of autophagic activity in comparison to its basal level under FM conditions. Importantly, this acceleration by ND was best revealed under conditions of BAF treatment, reflecting the previously reported fast turnover of formed autophagosomes (i.e. autophagic flux) [35]. The addition of BAF increased the vesicle size as well as the vesicle count of lysosomes, most notably for autolysosomes. Interestingly, BAF had no effect on the vesicle sizes of autophagosomes and lead to a reduction of autophagosome counts, presumably due to maintained autophagosome-lysosome fusion events [36], consistent with the elevated numbers of lysosomes and autolysosomes.

### Data-driven model parameter fitting for basal autophagic activity

In order to parameterize our model using single-cell measurements we created a fitness function (described in Materials and methods), which was minimized via a fitting procedure including a genetic algorithm, as outlined in Figure 1C. For each parameter set, the mean result of 100 simulations was calculated and compared to the biological data, in order to calculate a fitness value corresponding to the similarity between the mean model results and the biological data. The parameter set for the core model was simultaneously optimized for FM conditions, with and without BAF.

**Figure 4:**
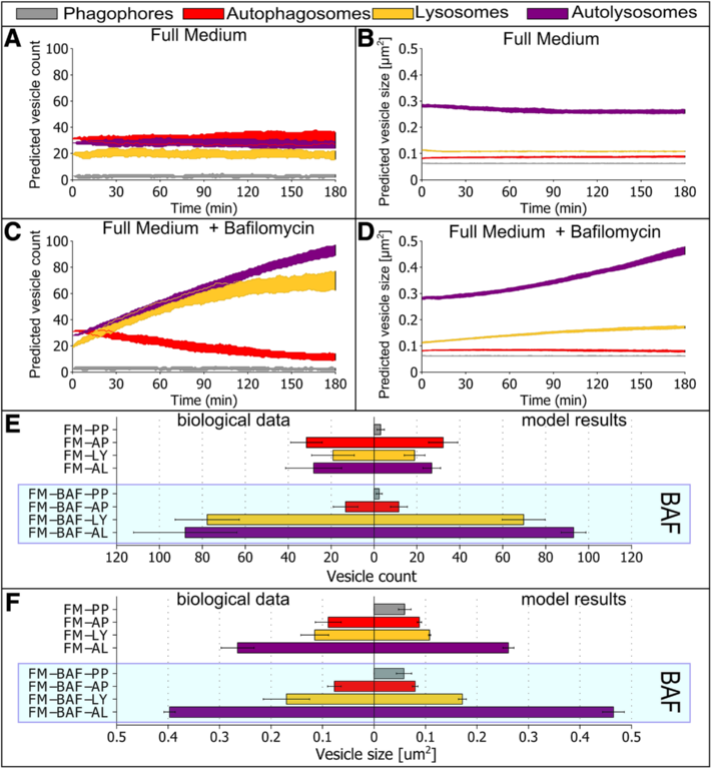
**Core model simulation of autophagic flux dynamics.** The core model was simulated for 100 runs under indicated conditions for 3 hours. For each agent the plotted shaded area corresponds to the 25 and 75 quantile of data. **A** Time course of vesicle count under FM conditions. **B** Time course of the vesicle size under FM conditions. **C** Time course of vesicle count under FM conditions with the addition of BAF. **D** Time course of the vesicle size under FM conditions with the addition of BAF. **E**-**F** Comparison of simulation results to experimentally determined values. **E** (Right side) Simulated mean vesicle count at the 3 hour time point, with and without the addition of BAF. (Left side) Experimentally measured results (from Figure 3). **F** (Right side) Simulated mean vesicle size at the 3 hour time point, with and without the addition of BAF. (Left side) Experimentally measured results (from Figure 3). The blue shaded box indicates conditions in the presence of BAF.

To compare the accuracy of the fit for the best-found parameter set, the fitness values of 200 randomly chosen parameter sets (every parameter was chosen from a specified range of values) were calculated and the results are shown in Table 1. Results demonstrate that the best-found parameter set was significantly better than a randomly chosen parameter set. The best-found parameter set is detailed in Table 2. The simulated time course results for each of the four types of agents from 100 runs of the best-found parameter set are shown in Figure 4A-D, while the mean and the standard deviation of the results after 180 minutes of simulation are shown in Figure 4E-F, with a direct comparison to the biological data (Figure 3).

**Table 1:** **Comparison of the best-found parameter set for the core model with 200 randomly generated parameter sets**

**Condition**	**Mean fitness of 200 randomly generated parameter sets**	**Best fitness of 200 randomly generated parameter sets**	**Fitness of the best parameter set**	**Average deviation of the best parameter set to the biological data**
FM	303783.2	6761.225	65.4	3.3%
FM + BAF	524681.5	4578.285	606.7	10.05%

**Table 2:** **Overview of the main used parameters for the core model**

**Parameter name**	**Value**
Initial number of phagophores	3
Initial size of phagophores	0.06 μm^2^
Creation rate of phagophores	0.42 min^-1^
Mean maturation time of phagophores	7.5 min
Growth rate of phagophores	6e-04 μm^2^ min^-1^
Initial number of autophagosomes	31
Growth rate of autophagosomes	8e-05 μm^2^ min^-1^
Initial number of lysosomes	19
Initial size of lysosomes	0.095 μm^2^
Creation rate of lysosomes	1.26 min^-1^
Mean life time of lysosomes	18 min
Growth rate of lysosomes	0.002 μm^2^ min^-1^
Initial number of autolysosomes	28
Initial size of autolysosomes	0.225 μm^2^
Mean life time of autolysosomes	95 min
Growth rate of autolysosomes	0.0012 μm^2^ min^-1^
Chance of reformation autolysosome to lysosome	50%
Rate of movement	2 μm min^-1^
Chance of autophagosomal movement towards nucleus	85%
Chance of lysosomal movement towards nucleus	55%
Chance of fusion between a non-autolysosome and an autolysosome	45%
Chance of fusion between two autolysosomes	15%

Initial number of autophagosomes, lysosomes and autolysosomes were chosen according to biological data (Figure 2), all other parameters were chosen according to the parameter fitting.

### Limitations to parameter fitting identifies that inhibition of lysosomes through BAF rapidly suppresses mTOR

While our simulation results were consistent with measured basal autophagy activities, parameter fitting was not sufficiently optimized for conditions of lysosomal inhibition. Compared to experimental measurements, the model predicts insufficient numbers of lysosomes and autophagosomes, and greater numbers of autolysosomes (Figure 4E-F). This disagreement between simulation and experimental observations suggested additional biological mechanisms and/or regulatory steps were not present in our model. Interestingly, it was recently proposed that inhibition of lysosomal function may decrease mTOR activity [37]. As performed here, autophagic flux measurements commonly involve the comparison of autophagy measures under the (mostly short-term) presence versus absence of lysosomal inhibitors [32]. As such, reduced mTOR activity by lysosomal inhibition could influence autophagic flux measurements. While the short-term effect of lysosomal inhibition on mTOR activity state is undetermined, it has indeed been shown that prolonged (overnight to 24 hours) lysosomal inhibition by BAF [38] or the lysosomotropic agent chloroquine [16] results in mTOR inactivation. Importantly, such inhibition of mTOR could be responsible for an enhancement of autophagosomal [39] and lysosomal formation [16,40,41], and, together with the known positive influence of mTOR on autolysosomal reformation (ALR) [42,43], could explain the difference between our model predictions and experimental observations.

**Figure 5:**
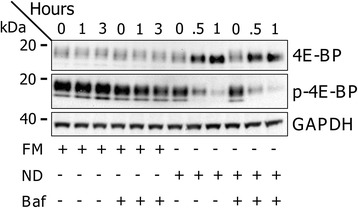
**Bafilomycin A1 decreases mTOR activity under FM and ND conditions.** HeLa cells were submitted to FM and ND conditions, in the absence and presence of BAF (100 nM). Western blot analysis was performed with protein samples taken during time periods of 0-3 hours (FM) and 0-1 hours (ND). Antibodies against 4EBP1 and phosphorylated 4EBP (p-4EBP1) were used to detect mTOR activity. An antibody against GAPDH was used as loading control.

We therefore measured the effect of short-term lysosomal inhibition by BAF on mTOR activity, under basal and activated autophagy conditions. HeLa cells were submitted to FM or ND conditions, with or without 100 nM BAF for the indicated time periods. To monitor mTOR activity, levels and phosphorylation state of its target, the translation repressor 4E-BP1 [32], were analyzed by Western blot (Figure 5). While levels of 4E-BP1 and T37/46 phosphorylated 4E-BP1 (p-4E-BP1) were stable in FM conditions, treatment of cells with BAF under FM conditions decreased p-4EBP1 within 1 hour. Under ND conditions, levels of p-4E-BP1 were decreased at 0.5 hours, and further decreased at 1 hour. Treatment with BAF under ND conditions further enhanced this decrease in p-4E-BP1 at both 0.5 and 1 hour. Of note, total levels of 4E-BP1 increased under FM in response to BAF, under ND alone, and further with ND/BAF, in accordance with degradation of phosphorylated 4E-BP1 [44].

These experimental data demonstrate that BAF acts rapidly to suppress mTOR on a time scale of minutes to hours. Thus, together, experimental and simulation results reveal the need to control for the impact of BAF-mediated lysosomal inhibition on mTOR activity, and to determine if a correction is required for experimental determination of autophagic flux.

### Realistic simulations of autophagy dynamics require integration of nutrient uptake and recycling

**Figure 6:**
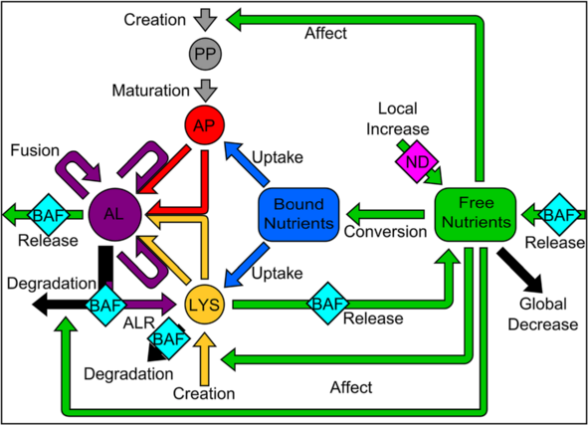
**Overview of the integrative model of autophagy.** Schematic describing interactions of the four agent types from Figure 1, expanded by the addition of nutrients. Each agent is color-coded; phagophores (PP) in grey, autophagosomes (AP) in red, lysosomes (LYS) in yellow and autolysosomes (AL) in purple. Two distinct nutrient-type global values are included: bound nutrients (blue) and free nutrients (green). Merging arrows represent fusion events between the two agents to form or expand an autolysosome. Black arrows represent degradation events. Arrows marked with BAF or ND indicates that this pathway is influenced under conditions with BAF or by ND. Black arrows represent degradation events.

The above findings illustrate the fundamental relationship between metabolic signaling and autophagy activities. We therefore implemented a metabolic function for autophagy, by including an environmental source for and autophagy-mediated turnover of nutrients (Figure 6, NetLogo model file as Additional file 2). The cellular nutrient status was defined as a combination of two distinct nutrient-type global values. The first was denoted as free nutrients, representing amino acids and other basic biochemical building blocks which are not in this form targeted by autophagosomes. The second was denoted as bound nutrients, representing proteins and other macromolecules which can be taken up and degraded by autophagy. Anabolic events are represented by free nutrients undergoing a conversion to bound nutrients at a parameterized rate. Catabolic events are simulated as two events corresponding to (i) non-macroautophagy lysosomal degradation processes (e.g. [45]), which are regulated by lysosomal conversion of bound nutrient back to free nutrient at a parameterized rate, and (ii) release of degraded autophagy substrates during degradation of the autolysosome. We assumed that the amount of free nutrients released by degrading autolysosomes was equivalent to the amount of bound nutrients consumed by its precursor autophagosomes (Table 3).

**Table 3:** **Overview of the effect on nutrients in the integrative model**

**Effect**	**Free nutrients**	**Bound nutrients**
Local increase of free nutrients	+	0
Global decrease of bound nutrients	0	-
Global conversion of free to bound nutrients	-	+
Lysosomal recycling	+	-
Autophagosomal uptake	0	-
Autolysosomal release	+	0

(+) indicates an increase, (-) indicates a decrease, (0) indicates no change.

In the core autophagy model (Figure 1), we considered the creation rate of phagophores and lysosomes, the degradation rate of autolysosomes, and the lysosomal positioning in the cell, to be independent parameters. To couple autophagy with metabolic state, we subsequently integrated these parameters with the environmental level of free nutrients. In this integrative model (Figure 6), low levels of free nutrients increase the creation of phagophores and lysosomes and reduce degradation of autolysosomes. Further, as lysosomal positioning is dependent on available free nutrients [14,15], low levels of free nutrients reorders lysosomes towards the nucleus.

For this integrative model a different parameter fitting strategy was employed, consisting of two independent steps. As an initial step, the model was fit to FM conditions, and a good parameter set with a fitness value of 81.3 was obtained. On average the integrative model differed less than 4% from the biological data. Next, with this initial parameter set, the best fit was determined for the effect of BAF, ND, and the combination of both on the creation rate of phagophores and lysosomes, on the degradation of autolysosomes, and on lysosomal positioning. The best fit results, including the resulting amount of free nutrients in the cell after 180 minutes of simulations, are shown in Table 4.

**Table 4:** **Results of the fitting process for the integrative model**

	**Relative creation rate phagophores**	**Relative creation rate lysosomes**	**Relative degradation rate autolysosomes**	**Relative lysosomal positioning**	**Resulting difference free nutrients**
FM	1	1	1	0%	0
FM + BAF	1.61	1.2	0.1	+ 2.2%	4.4
ND	4.86	2.98	12.04	+ 5.25%	10.5
ND + BAF	6.63	3.6	2.61	+ 8.55%	17.1

The relative change of the creation rate of phagophores and lysosomes, the degradation rate of autolysosomes and of lysosomal positioning for FM with BAF and ND with and without BAF which obtain the best fitness values are shown in comparison to FM conditions.

A good fitness value resulted in increased autolysosomal degradation under ND conditions, as expected. However, compared to FM conditions, under ND conditions with the addition of BAF, a higher rate of autolysosomal degradation was needed in order to obtain a good fit, suggesting that a basal level of lysosomal function occurs under BAF conditions. The mode of action for BAF is to inhibit V-ATPase-mediated acidification of the lysosomal lumen [31]. Thus, this prediction is plausible, as lysosomal hydrolases are maximally active at low pH, but maintain some functionality at neutral pH [46]. Furthermore, the direction of change in the creation rate of phagophores and lysosomes was as expected, as all three tested conditions showed an increased production of these two agents, with an increase correlating with the difference in the free nutrients.

Importantly, lysosomal positioning in response to the availability of free nutrient levels is crucial for the activation and the fusion processes of autophagy [14,15]. To link the change of rates and positioning with the change in free nutrient levels, a function of the following formula was fit to the data obtained from the fitting procedure (Table 4):

$$ linear\_ factor* delta\_ nutritio{n}^{exponential\_ factor}+1 $$

The best results for simulating autolysosome degradation were obtained by allowing BAF to reduce the degradation by a factor of 20. Of note, this substantial deceleration was partly reversed by the increase of degradation in response to the lack of free nutrients, so that the measured values, as shown in Table 4, were reached. These fitted functions were then implemented in the integrative model, and the mean output for this parameter set was calculated for 100 simulations.

**Figure 7:**
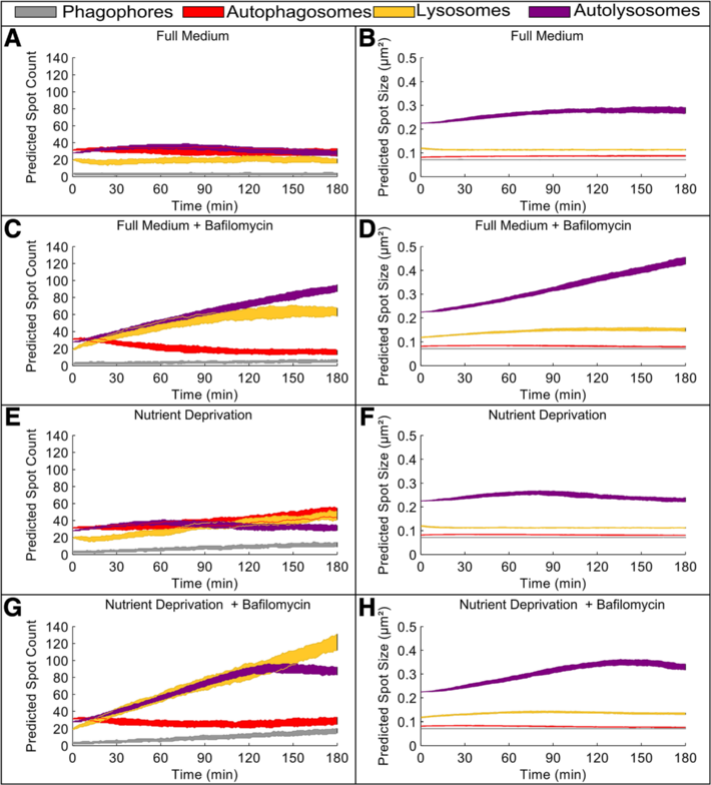
**Optimized integrative model simulation of autophagic flux dynamics.** The optimized integrative model was simulated for 3 hours, 100 times for each condition. For each agent the plotted shaded area corresponds to the 25 and 75 quantile of data. **A** Vesicle count under FM conditions. **B** Vesicle size under FM conditions. **C** Vesicle count under FM conditions with BAF. **D** Vesicle size under FM conditions with BAF. **E** Vesicle count under ND conditions. **F** Vesicle size under ND conditions. **G** Vesicle count under ND conditions with BAF. **H** Vesicle size under ND conditions with BAF.

**Figure 8:**
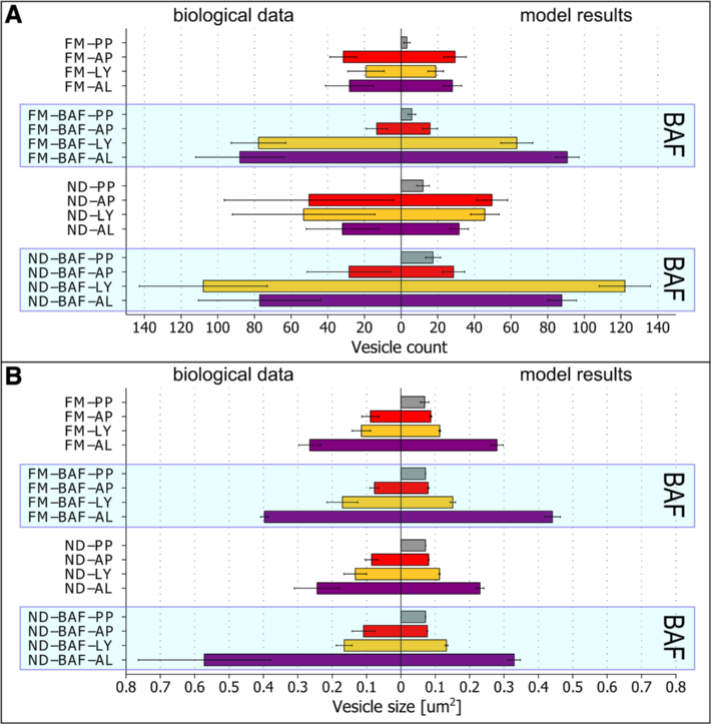
**Comparison of optimized integrative model simulation results to biological measurements.** The integrative model was simulated under indicated conditions for 3 hours, and results for the time point of 180 minutes are shown. The left side corresponds to biological measurements from Figure 3, and the right side indicates simulation results. **A** Mean vesicle count with standard deviation for the four different conditions. **B** Mean vesicle size with standard deviation for the four different conditions. The blue shaded box indicates conditions in the presence of BAF.

As an index to evaluate the accuracy of this best-found parameter set, its fitness value was compared to the fitness values of 200 randomly generated parameter sets (Table 5). Our best obtained fit, based on 100 measurements, showed 14-fold higher accuracy than the randomly generated parameter set. Moreover, the integrative model including these fitted functions closely resembles the biological data, with a difference less than 4% for FM conditions. The time courses from 100 runs for the first 180 min of the best parameter set are shown in Figure 7, and the mean results for the time point t = 180 min are shown in Figure 8, Of note, the high standard deviation indicates a high degree of cell-to-cell variability in our simulations. This is further demonstrated in histograms of the modeling results at t = 180 min for each of the four conditions (Additional files 3, 4, 5 and 6). An overview of the best-found parameter set is shown in Table 6.

**Table 5:** **Comparison of the best-found parameter set for the integrative model with 200 randomly generated parameter sets**

**Condition**	**Mean fitness of 200 randomly generated parameter sets**	**Best fitness of 200 random generated parameter sets**	**Fitness of the best parameter set**	**Average difference of the best parameter set to the biological data**
FM	14136473	107185.2	81.3	3.68%
FM + BAF	1279520	138923.1	996.9	12.88%
ND	2301350	107662.4	494.8	9.08%
ND + BAF	717115.8	117476.8	3415.4	23.85%

**Table 6:** **Overview of parameters used for the integrative model**

**Parameter name**	**Value**
Initial number of phagophores	3
Initial size of phagophores	0.07 μm^2^
Creation rate of phagophores	0.44 min^-1^
Linear nutrient factor creation phagophores	0.206
Exponential nutrient factor creation phagophores	1.179
Mean maturation time of phagophores	7.5 min
Growth rate of phagophores	5e-04 μm^2^ min^-1^
Initial number of autophagosomes	31
Growth rate of autophagosomes	8e-05 μm^2^ min^-1^
Initial number of lysosomes	19
Initial size of lysosomes	0.1 μm^2^
Creation rate of lysosomes	1.23 min^-1^
Linear nutrient factor creation lysosomes	0.112
Exponential nutrient factor creation lysosomes	1.124
Mean life time of lysosomes	18 min
Growth rate of lysosomes	0.0021 μm^2^ min^-1^
Initial number of autolysosomes	28
Initial size of autolysosomes	0.225 μm^2^
Degradation rate autolysosomes	0.15 min^-1^
Linear nutrient factor degradation autolysosomes	0.0069
Exponential nutrient factor degradation autolysosomes	3.14
Growth rate of autolysosomes	0.00065 μm^2^ min^-1^
Chance of reformation autolysosome to lysosome	50%
Rate of movement	2 μm min^-1^
Chance of autophagosomal movement towards nucleus	85%
Chance of lysosomal movement towards nucleus basis	55%
Chance of lysosomal movement towards nucleus via nutrient status	+0.5% DifferenceNutrition^-1^
Chance of fusion of a non-autolysosome and an autolysosome	45%
Chance of fusion of two autolysosomes	30%
Mean free nutrients for initialization of the model	20
Mean bound nutrients for initialization of the model	20
Diffusion of free nutrients	70%
Diffusion of bound nutrients	50%
Local increase of free nutrients	1.1 min^-1^ borderpatch^-1^
Global decrease of free nutrients	0.05 min^-1^ patch^-1^
Global conversion of free nutrients to bound nutrients	0.045 min^-1^ patch^-1^
Lysosomal conversion of bound nutrients to free nutrients	1.5 min^-1^ lysosome^-1^
Autophagosomal uptake of bound nutrients	2.25 min^-1^ autophagosome^-1^
Autolysosomal release of free nutrients	2.25 min^-1^ autolysosome^-1^
Effect BAF on the degradation rate of autolysosomes	Degradation rate AL × 0.05

Initial number of autophagosomes, lysosomes and autolysosomes were chosen according to our biological data, all other parameters were chosen according to the parameter fitting.

### High accuracy of dynamic simulations with the integrative autophagy-environmental model

As expected, under FM conditions the model predicted a near steady-state vesicle count and vesicle size (Figure 7A-B). The addition of BAF under FM conditions increased the number of phagophores over time, due to reduce free nutrient availability (Figure 7C-D). After ~120 minutes the vesicle count of autophagosomes and lysosomes reached a steady state, while the number of autolysosomes continued to increase. This result is also observed in the change of vesicle sizes.

Under ND conditions (activated autophagy) the number of phagophores, autophagosomes and lysosomes increased over time, while the number of autolysosomes reached a maximum at ~ t = 90 min and decreased thereafter, but at levels always higher than the starting value (simulated to t = 180 min) (Figure 7E-F). Under ND conditions, the addition of BAF resulted in increased phagophores and lysosomes over time (Figure 7G-H). The number of autophagosomes initially decreased, reaching a minimum at ~ t = 100 min, followed by a slight increase thereafter. The number of lysosomes and autolysosomes underwent a near linear increase in the first 120 min, but after 120 min the number of autolysosomes decreased while the number of lysosomes continue to increase. For each of the four conditions, a 3-hour simulation movie, with the corresponding time courses is included in the supplementary information (Additional files 7, 8, 9 and 10).

In comparison to the high-resolution imaging results, the integrative model showed accurate results for the vesicle count under all four conditions (Figure 8A). However, vesicle sizes were partly inconsistent (Figure 8B). This was most pronounced for ND conditions, where vesicle sizes are inferior to experimentally determined values. This deviation of the model results for ND conditions indicates, that the growth rates of all four agents are increased under ND conditions in the cell and should therefore also be linked to the level of free nutrients.

### Emergent spatial patterns of autophagic vesicles match high-resolution, single-cell images

**Figure 9:**
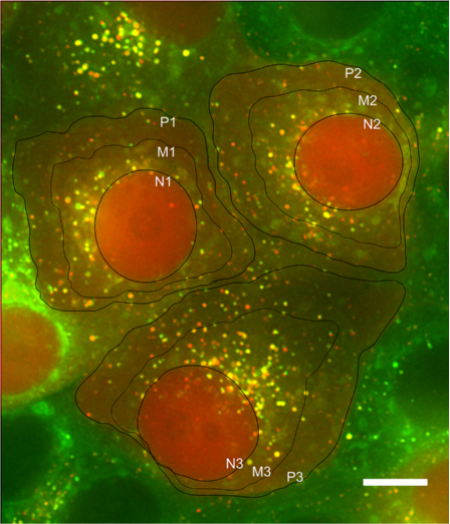
**Nuclear clustering of autophagosomes, lysosomes and autolysosomes under ND conditions.** MCF7 cells stably expressing GFP-Rab7 and mCherry-LC3, submitted to 3 hours of ND. N designates the bounded nuclear region, and P corresponds to the periphery of the cell. The M (perinuclear) region was obtained by partitioning the cell at an equal distance between the N and P lines. Numbers are cell identifiers. Scale bar, 10 μm.

A major advantage of ABM is the visualization of dynamic behavior, which can be directly compared to experimental results. We remarked that the graphical output of our model demonstrated an obvious peri-nuclear clustering of autophagosomes and lysosomes. Interestingly, this phenotype was observed in our cell imaging experiments (Figures 3 and 9). Cellular partitions in Figure 9 identify nuclear (N), perinuclear (M) and cell periphery (P) regions. The subcellular clustering of vesicles in the M regions was most pronounced under ND conditions (Figure 9), likely due to a combination of increased numbers of autophagosomes and lysosomes and reordering of lysosomes towards the nucleus.

### Impact of minor alterations to vesicle positioning and nutrient levels on long-term behavior of autophagy

In the above, simulated basal autophagy conditions maintained a pseudo-steady-state and short-term perturbation simulations were highly accurate. We subsequently sought to determine the effect of minor influences on vesicle transport by long-term emergent behavior. We simulated changes to dynein motor protein activities, which transports vesicles along microtubules towards the nucleus [47,48]. Impaired vesicle transport contributes prominently to neurological diseases [49], and functionally arises from alterations in bi-directional transport control. There are many mutations reported which lead to an impaired vesicle transport, with a different level of severity ranging from little effects to near total abolishment [50].

**Figure 10:**
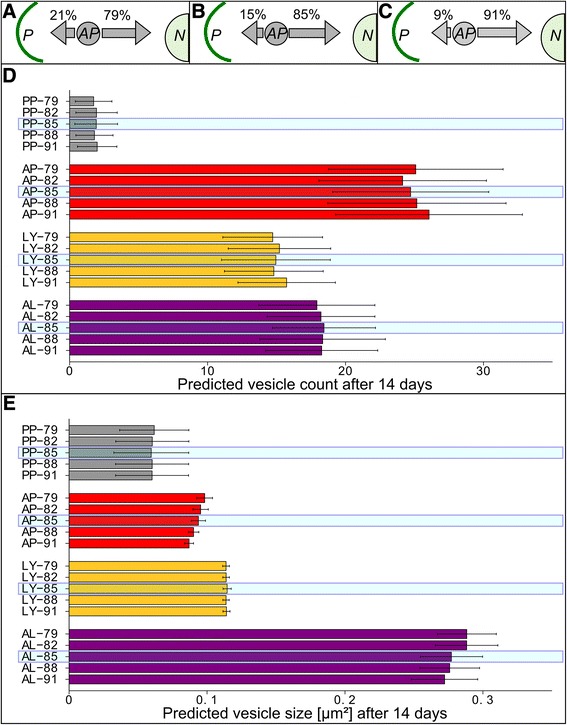
**Impact of minor changes to autophagosome movements in long-term simulations of optimized integrative model. A**-**C** Schematic describing bi-directional movement of autophagosomes (AP) between the cell periphery (P) and nucleus (N). Arrows denote probabilities for AP agent movements. **D**-**E** 100 Simulations corresponding to 14 days (20160 min) were performed, using indicated altered transport probabilities. Steady-state vesicle dynamics at 14 days are reported. **D** Mean vesicle count with standard deviation. **E** Mean vesicle size with standard deviation. Numbers on the y-axis represent the probability of autophagosome movement towards the nucleus, centered on the standard value of 85% (indicated by the blue shaded box).

We therefore determined the result of a range (+/- 3 and 6%) change in the probability of autophagosome movement towards to the nucleus, and simulated 14 days (Figure 10). With decreasing transport towards the nucleus, the size of autophagosomes and autolysosomes increased over time, and vesicle positioning at the nucleus decreased. The increased size of autophagosomes and autolysosomes indicates reduced autophagic flux, consistent with impaired dynein transport [51].

As a second approach, we investigated emergent behavior stemming from metabolic perturbations, which contributes to different diseases and impacts autophagy [23]. We simulated the effect of 2.5% and a 5% increase/decrease in cellular nutrient uptake for 14 days (Additional file 11). With decreased nutrient uptake, the vesicle number for all agents increased rapidly. The size of lysosomes and autolysosomes showed no change, while a minor decrease in autophagosomes size and an increase in phagophore size was observed. These changes represent the change from FM towards ND conditions, which showed a similar pattern. With increased nutrient uptake vesicle numbers for all agents decreased, while the sizes of agents was altered heterogeneously; phagophore size was reduced, autophagosome and autolysosome sizes increased, and lysosome size remained constant. We further simulated the effects of a 2.5% and 5% increase/decrease in cellular metabolism (Additional file 12), hence in the conversion rate from free nutrient to bound nutrient. Since a lower nutrient uptake rate and a higher nutrient conversion rate both have the same decreasing effect on overall free nutrient levels in the cell, these results show a similar pattern to the results obtained from a decreased nutrient uptake.

## Discussion

In this study, we established an ABM for the core processes of autophagy. Through parameter fitting of measured autophagy activities we were able to accurately simulate spatio-temporal dynamics of basal and activated autophagic flux. Moreover, during model optimization, the inability to obtain good fits from experimental measurements indicated that the initial, core model design, focusing on pathway dynamics, required integration of the autophagic process with the cellular nutrient levels. To that end, we integrated a regulatory control of nutrient levels on autophagy pathway events with autophagy recycling of cellular components. We present the application of our model for investigating autophagy in a short time scale, i.e. minutes to hours, and at extended time scales, i.e. days to weeks.

A major benefit to pathway modeling is the ability to predict dynamics which may be difficult or impossible to observe experimentally, but also to suggest novel experiments based on inaccurate model behaviors. Indeed, a direct result of initial model limitations was the prediction and experimental validation that BAF-induced lysosomal inhibition results in a rapid reduction of mTOR activity. This is in accordance with a recently proposed mechanism [37], and presumably due to reduced protein degradation and amino acid release [11,12]. This suppressive effect could account for insufficient numbers of autophagosomes and lysosomes, as mTOR acts as a negative regulator of lysosomal biogenesis [16,40,41] and autophagosome formation [39].

Furthermore, ABM offers the advantage of allowing direct comparison of simulated spatially-resolved dynamics to experimental datasets. The rule set employed here resulted in a phenotype with autophagic vesicles concentrating in the peri-nuclear region. Upon further examination of our image dataset we recognized the remarkable similarity between simulated localization of autophagic vesicles and experimental observations.

The resulting ABM model not only realistically captured short-term dynamic behavior, but also provided a novel tool to predict long-term system evolution. As a proof-of-principle we altered the influences of vesicle movements and environment on autophagy. By decreasing the probability of autophagosome transport towards the nucleus, we predicted measurable impact on autophagy and vesicle distribution emerging over weeks. Further, increasing the nutrient source had the most obvious effect of altering vesicle sizes. These results emphasize the importance of including spatial regulation and environmental influences, and demonstrate the possibility to investigate dynamics otherwise could not be monitored at an equivalent spatial and/or temporal resolution experimentally.

Previously, Martin et al. used ODE modeling to perform the first systems biology investigation of autophagosome dynamics [18]. The authors predicted dynamic deterministic and stochastic autophagy pathway behavior consistent with experimental measurements. In addition, Tavassoly et al. presented an ODE approach, where through modeling crosstalk between autophagy and apoptosis signaling pathways the authors were able to predict dynamic autophagy and cell death responses to metabolic and calcium stresses [19]. However, in both studies, these ODE approaches assumed a ‘mixed-bag’, homogenous distribution of pathway components, and were as such limited to integrating and reporting concentration changes over time. Here we demonstrate that spatial-temporal modeling allows for full integration of rich, complex phenotypes from imaging datasets, as well as high-content, qualitative knowledge from literature. Furthermore, using ABMs, inherent phenotypic variability arises due to probabilities of interactions among heterogeneously distributed agent populations, which corresponds more directly to the emergence of sub-cellular and cellular heterogeneities [52]. Points of crosstalk between apoptosis and autophagy underlie the cell fate decision [19,53]. To address such crosstalk future work will integrate rules for crosstalk between autophagy and apoptosis agents, including pro-apoptotic mitochondrial autophagy receptors [9]. Furthermore, higher accuracy will be achieved through the use of additional GFP-based biosensors for autophagy, including 2xFYVE [54], pH-sensitive tandem sensors for quantifying transitions between autophagosomes and autolysosomes [26,55], and single-cell, spatio-temporally correlated autophagy and apoptosis data-sets (e.g. [56]).

## Conclusion

Here, we developed an ABM to compare and integrate spatio-temporal simulations of autophagy with experimental data, and to predict non-intuitive findings. The resulting model captures with high accuracy short-term and long-term behaviors, and through the use of NetLogo, is available as a community resource, e.g. to further integrate and investigate regulation stemming from pathway crosstalk with apoptosis and specific forms of autophagy.

## Materials and methods

### Modeling

ABMs were developed using the open source toolkit NetLogo (v5.0) [29]. Statistical analyses and plotting was performed using the open source environment for statistical computing R [57] in combination with the R-package RNetLogo [58,59]. Fitting of the biological data was performed through minimization of a predefined fitness function in combination with a genetic algorithm provided by the R package GA [60], and a parallelized implementation of the model controlled via RNetLogo.

### Fitness evaluation of parameter sets

To calculate the fitness of the model, the predicted vesicle count and vesicle size of autophagosomes, lysosomes and autolysosomes, were compared with the corresponding biological data by using the following formula:

$$ Fitness\left[ Model\right]={\displaystyle \sum_{i,j}^{\begin{array}{c}\hfill i\ \in\ Count,\kern0.5em  Size\hfill \\ {}\hfill j\ \in\ AP,\  LY,\  AL\hfill \end{array}}}{\left(\left(\left(\ \frac{result\_ model\left(i,j\right)}{result\_ biological\left(i,j\right)}\right)-1\right)*100\right)}^2 $$

An exact match between model predictions and biological data would yield a fitness value of 0. Non-zero fitness values increase as a function of an increased difference between the model and the biological data. A 10% difference between the model and the biological data in one of the 6 different data points will result in a fitness value of 100.

We considered that a model predicting a 10% difference in two data points is a better model than one which shows a 20% difference in one data point, therefore the squaring was included in this formula, so that the first situation would obtain a fitness value of 200, while the second would obtain a fitness value of 400. The model is aborted automatically if the cell runs out of nutrients or if the total number of agents is higher than 500. In this case an extra penalty of 100000 was added to its calculated fitness value.

### Chemicals and antibodies

BAF was obtained from Enzo Life Sciences. Cell culture reagents were purchased from Invitrogen, Sigma Lonza and Pan Biotech. PhosSTOP phosphatase inhibitor and complete EDTA-free protease inhibitor were purchased from Roche Applied Science. RIPA buffer was obtained from Millipore. Antibodies were against GAPDH (Santa Cruz, #sc-25778), 4E-BP1 (Cell Signaling, #9452) and Phospho-4E-BP1 (Thr37/46) (Cell Signaling, #9459). Horseradish peroxidase (HRP)-conjugated secondary antibodies obtained from Genetex.

### Plasmids

GFP-RAB7 [33] and mCherry-LC3B [26] were previously described.

### Cell culture

Human MCF7 (Cell Line Services, Heidelberg) and HeLa Kyoto [61] cancer cell lines were maintained in FM consisting of DMEM supplemented with 10% FBS, L-glutamine, non-essential amino acids, penicillin, streptomycin and amphotericin B. ND was introduced using glucose-containing Hank’s Balanced Salt Solution (HBSS; Invitrogen # 14025-050).

### Western blotting

Protein samples were electrophoresed using Bis-Tris NuPage gels (Invitrogen) and transferred using the iBlot dry blotting system (Invitrogen). Membranes were blocked and incubated at 4°C overnight with primary antibodies, followed by incubation with HRP-coupled secondary antibodies. Membranes were then developed using a chemiluminescent substrate and a chemiluminescent imager (Intas). Four independent experiments were performed from which one representative blot is shown.

### Imaging

Cells were plated in microscopy μ-slides (iBidi). Widefield fluorescence microscopy was performed with a DeltaVision RT microscope system (Applied Precision) using a 60X oil immersion objective. Integrated stacks were captured using the OAI (optical axis integration) function. Images were deconvolved (Softworx).

### Image analysis

The image analysis was performed with the image processing package Fiji [62]. After a rolling ball background subtraction a threshold was manually applied to the images. Afterwards an overlay image of both channels was created and vesicle count, vesicle size and vesicle type (only GFP, only mCherry, GFP and mCherry) were analyzed.

## Additional files

Additional file 1:
**Core model of Autophagy.** NetLogo File for the core model of Autophagy, load and use with NetLogo 5.0.5 or newer. Additional file 2:
**Integrative model of Autophagy.** NetLogo File for the integrative model of Autophagy, load and use with NetLogo 5.0.5 or newer. Additional file 3:
**Cell-to-cell variability of the integrative model under FM conditions.** Histogram for the count and size [μm^2^] of each of the four agents of 100 simulations after 180 minutes. Additional file 4:
**Cell-to-cell-variability of the integrative model under FM conditions with BAF.** Histogram for the count and size [μm^2^] of each of the four agents of 100 simulations after 180 minutes. Additional file 5:
**Cell-to-cell-variability of the integrative model under ND conditions.** Histogram for the count and size [μm^2^] of each of the four agents of 100 simulations after 180 minutes. Additional file 6:
**Cell-to-cell-variability of the integrative model under ND conditions with BAF.** Histogram for the count and size [μm^2^] of each of the four agents of 100 simulations after 180 minutes. Additional file 7:
**Time course movie of the integrative model under FM conditions.** This movie shows the time-course of one model run for 180 minutes of the integrative model of autophagy under FM conditions. On the left, a part of the output of the NetLogo model is shown, on the right the corresponding data for the whole model is shown. Additional file 8:
**Time course movie of the integrative model under FM conditions with BAF.** This movie shows the time-course of one model run for 180 minutes of the integrative model of autophagy under FM conditions with BAF. On the left, a part of the output of the NetLogo model is shown, on the right the corresponding data for the whole model is shown. Additional file 9:
**Time course movie of the integrative model under ND conditions.** This movie shows the time-course of one model run for 180 minutes of the integrative model of autophagy under ND conditions. On the left, a part of the output of the NetLogo model is shown, on the right the corresponding data for the whole model is shown. Additional file 10:
**Time course movie of the integrative model under ND conditions with BAF.** This movie shows the time-course of one model run for 180 minutes of the integrative model of autophagy under ND conditions with BAF. On the left, a part of the output of the NetLogo model is shown, on the right the corresponding data for the whole model is shown. Additional file 11:
**Impact of minor changes to nutrient uptake in long-term simulations of optimized integrative model.** 100 Simulations corresponding to 14 days (20160 min) were performed, using the indicated altered nutrient uptake. Steady-state vesicle dynamics at 20160 minutes are reported. A Mean vesicle count with standard deviation. B Mean vesicle size with standard deviation. A-B Numbers on the y-axis represent the change of nutrient increase in percent times 10, i.e. 1000 indicates for 100%, which is the standard value (indicated by the blue shaded box). Additional file 12:
**Impact of minor changes to nutrient conversion in long-term simulations of optimized integrative model.** 100 Simulations corresponding to 14 days (20160 min) were performed, using the indicated altered nutrient conversion. Steady-state vesicle dynamics at 20160 minutes are reported. A Mean vesicle count with standard deviation. B Mean vesicle size with standard deviation. A-B Numbers on the y-axis represent the change of nutrient conversion in percent times 10, i.e. 1000 indicates 100%, which is the standard value (indicated by the blue shaded box).
