# Supplementary figures and images for: Agent-based modeling of autophagy reveals emergent regulatory behavior of spatio-temporal autophagy dynamics
Source: Cell Commun Signal. 2014 Sep 10;12:56. doi: 10.1186/s12964-014-0056-8 (PMC4172826; doi:10.1186/s12964-014-0056-8)

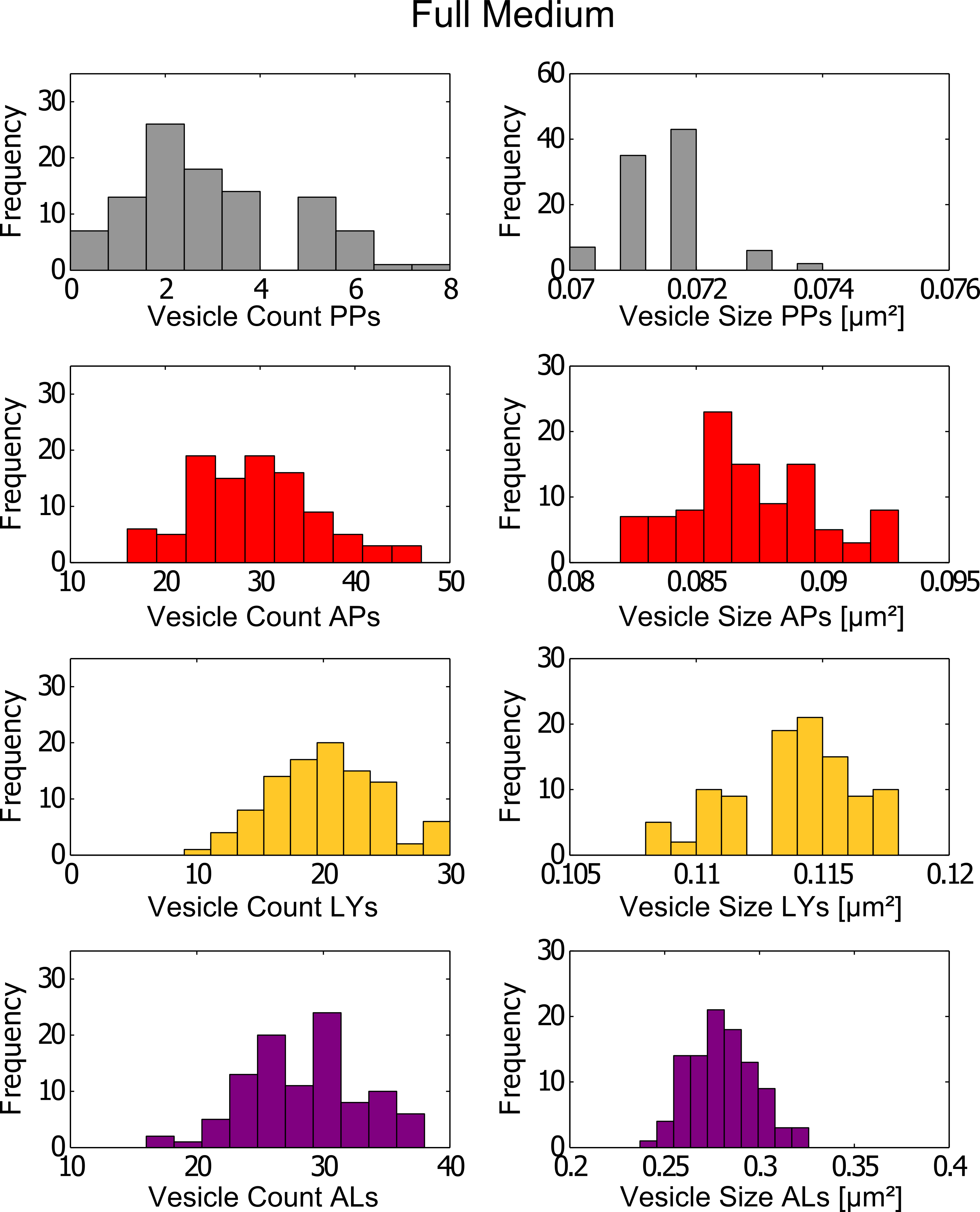

Supplement: Additional file 3: — Cell-to-cell variability of the integrative model under FM conditions. Histogram for the count and size [μm2] of each of the four agents of 100 simulations after 180 minutes. [file 12964_2014_56_MOESM3_ESM.png]

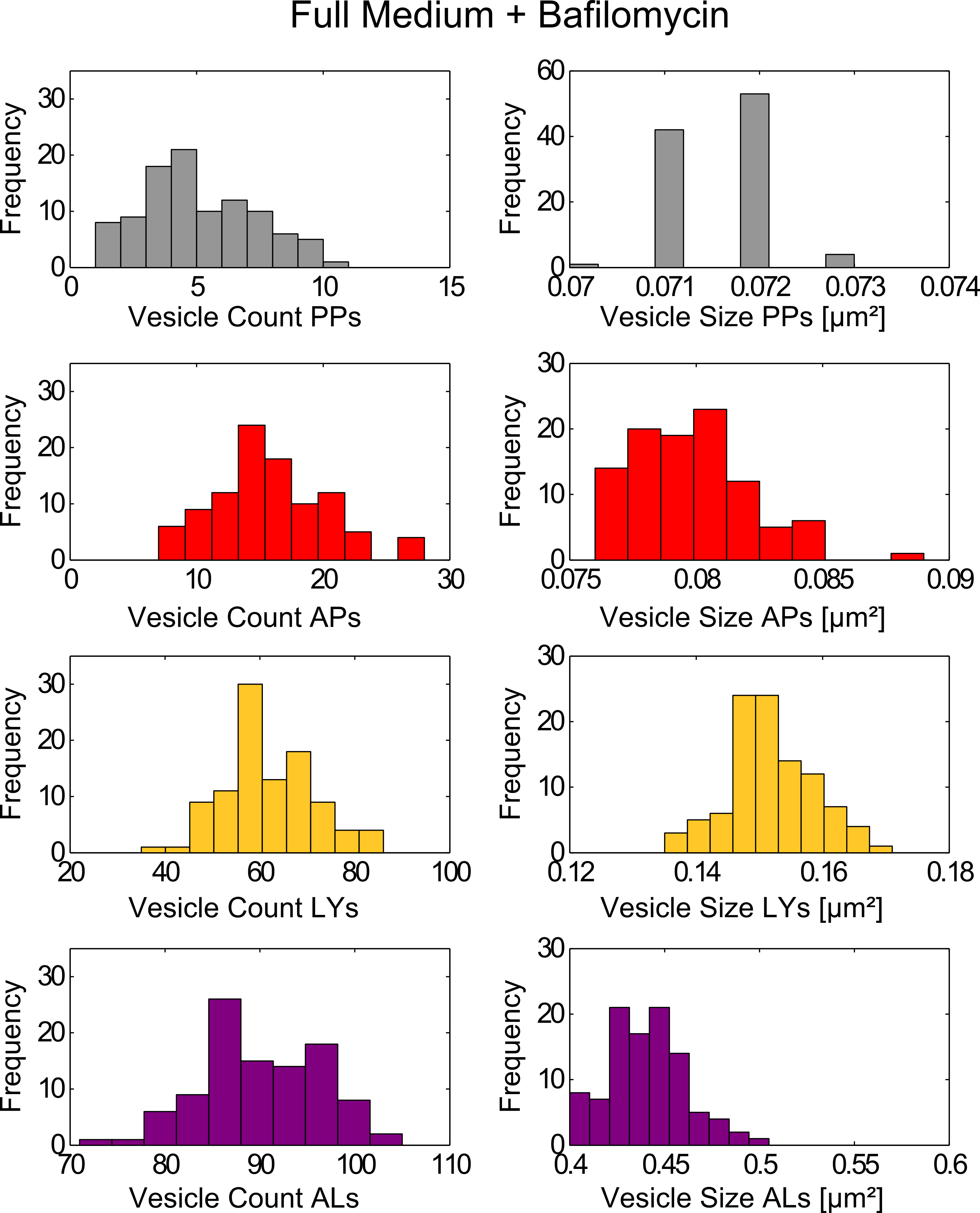

Supplement: Additional file 4: — Cell-to-cell-variability of the integrative model under FM conditions with BAF. Histogram for the count and size [μm2] of each of the four agents of 100 simulations after 180 minutes. [file 12964_2014_56_MOESM4_ESM.png]

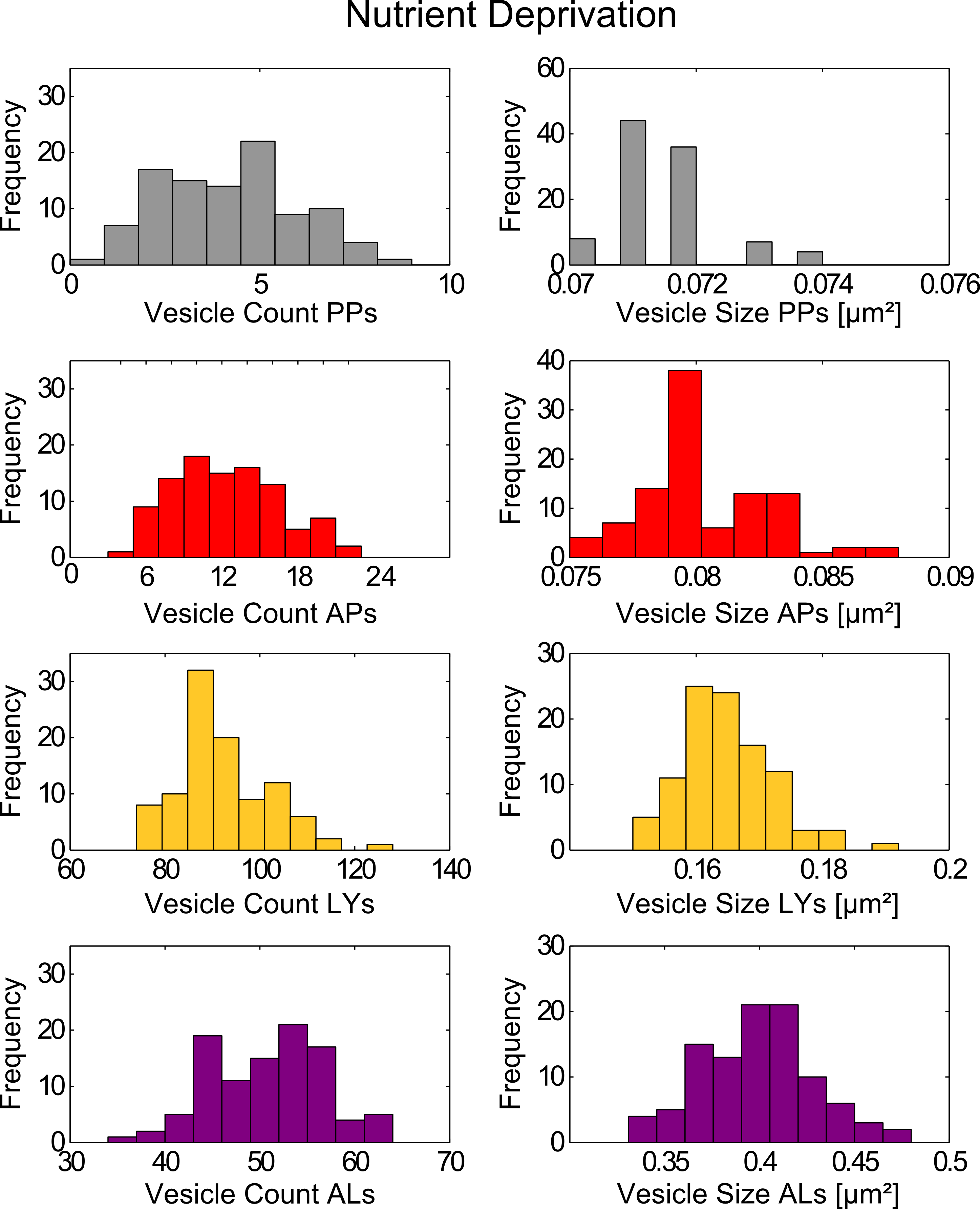

Supplement: Additional file 5: — Cell-to-cell-variability of the integrative model under ND conditions. Histogram for the count and size [μm2] of each of the four agents of 100 simulations after 180 minutes. [file 12964_2014_56_MOESM5_ESM.png]

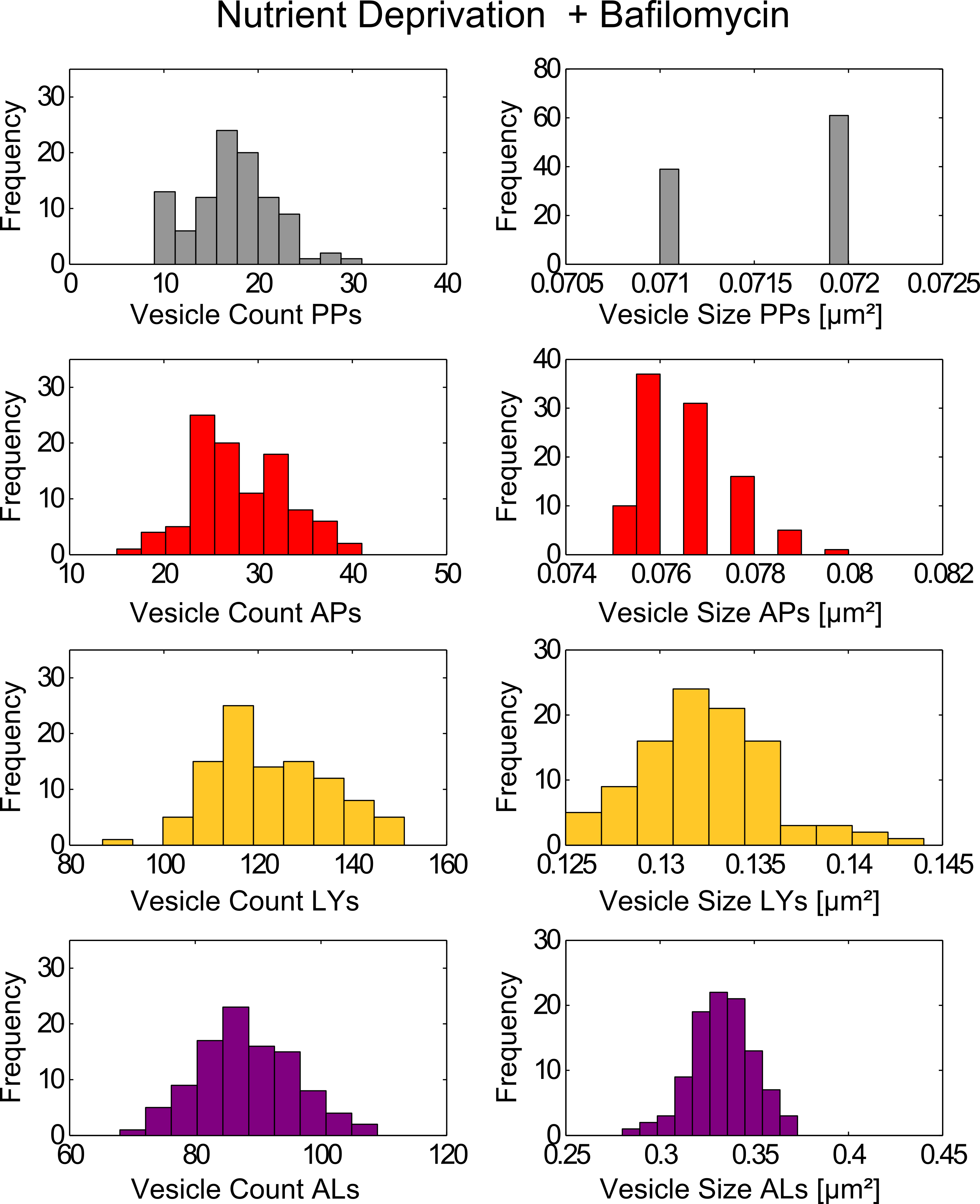

Supplement: Additional file 6: — Cell-to-cell-variability of the integrative model under ND conditions with BAF. Histogram for the count and size [μm2] of each of the four agents of 100 simulations after 180 minutes. [file 12964_2014_56_MOESM6_ESM.png]

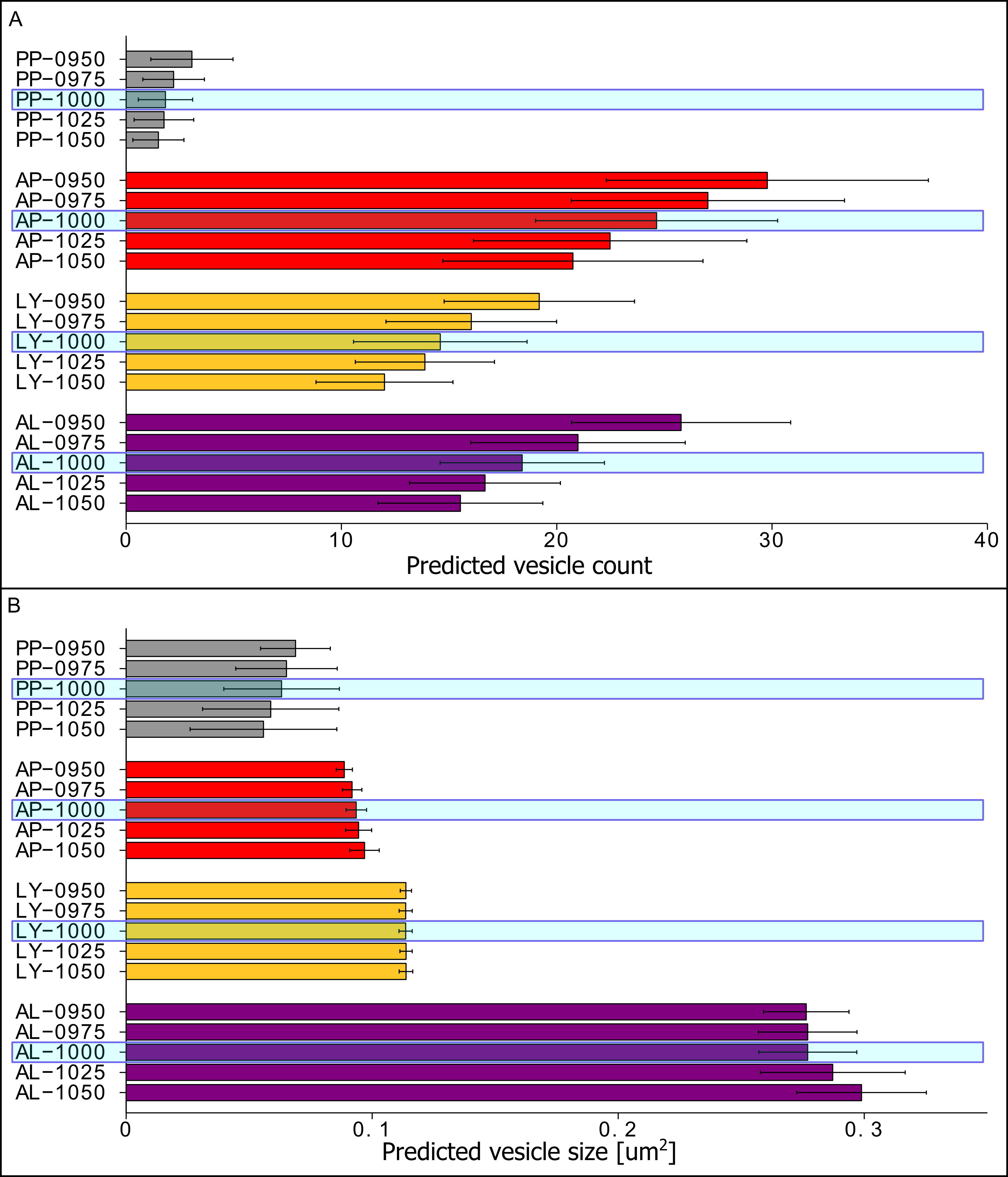

Supplement: Additional file 11: — Impact of minor changes to nutrient uptake in long-term simulations of optimized integrative model. 100 Simulations corresponding to 14 days (20160 min) were performed, using the indicated altered nutrient uptake. Steady-state vesicle dynamics at 20160 minutes are reported. A Mean vesicle count with standard deviation. B Mean vesicle size with standard deviation. A-B Numbers on the y-axis represent the change of nutrient increase in percent times 10, i.e. 1000 indicates for 100%, which is the standard value (indicated by the blue shaded box). [file 12964_2014_56_MOESM11_ESM.png]

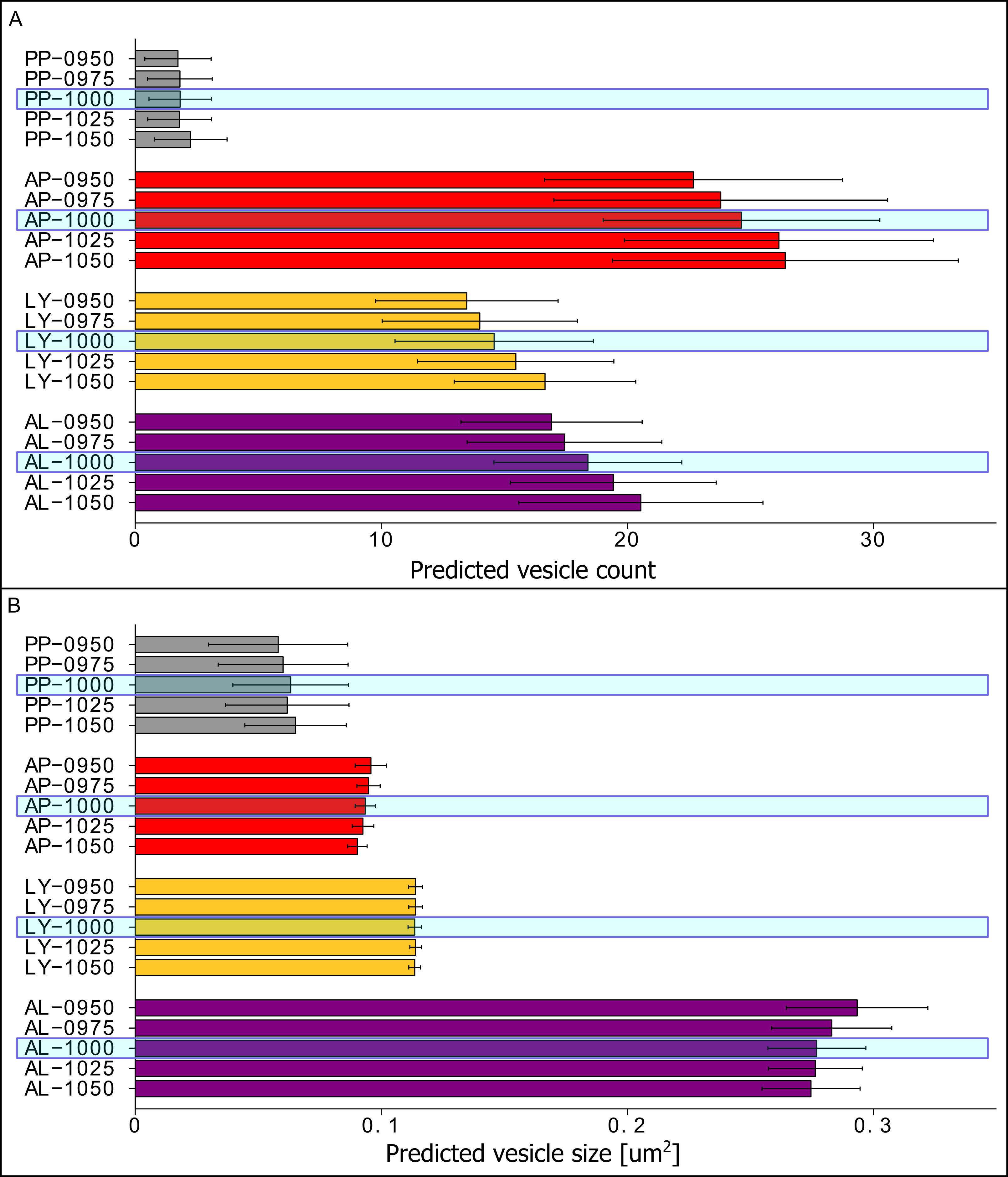

Supplement: Additional file 12: — Impact of minor changes to nutrient conversion in long-term simulations of optimized integrative model. 100 Simulations corresponding to 14 days (20160 min) were performed, using the indicated altered nutrient conversion. Steady-state vesicle dynamics at 20160 minutes are reported. A Mean vesicle count with standard deviation. B Mean vesicle size with standard deviation. A-B Numbers on the y-axis represent the change of nutrient conversion in percent times 10, i.e. 1000 indicates 100%, which is the standard value (indicated by the blue shaded box). [file 12964_2014_56_MOESM12_ESM.png]
